# Development of a high-speed imaging system for real time evaluation and monitoring of cardiac engineered tissues

**DOI:** 10.3389/fbioe.2024.1403044

**Published:** 2024-08-12

**Authors:** Antoine Belzil, Roselle Gélinas, Philippe Comtois

**Affiliations:** ^1^ Research Centre, Montreal Heart Institute, Montréal, QC, Canada; ^2^ Department of Pharmacology and Physiology, Universite de Montreal, Montréal, QC, Canada; ^3^ Institute of Biomedical Engineering, Universite de Montreal, Montréal, QC, Canada; ^4^ Laboratory of Genetics and Genomic Medicine of Inflammation, Montreal Heart Institute, Montréal, QC, Canada; ^5^ Department of Medicine, Universite de Montreal, Montréal, QC, Canada; ^6^ School of Pharmaceutical Sciences, University of Ottawa, Ottawa, ON, Canada

**Keywords:** functional imaging, CMOS, hiPSC cardiomyocyte, electrical stimulation, monitoring

## Abstract

Stem cell derived cardiac monolayers have high potential for tissue regeneration, *in vitro* drug testing and disease modeling. However, current differentiation protocols are still sub-optimal, resulting in cultures with variable yields and properties. We propose a high-speed lenseless imaging system, integrated with an electrical stimulation unit, to optimize the generation of these cultures. This tool relies on the variations of cellular patterns, during contraction, measured by digital imaging. The imaging system can monitor cardiac cell sheet function and structure, providing the necessary tools to quickly evaluate engineered monolayer. It can record high speed videos and capture high resolution images, from which tissue spatial organization and contractile characteristics can be obtained. Validation of the system was performed using cardiomyocytes derived from human induced pluripotent stem cell and neonatal rat cardiomyocytes. The imaging system allows the observation, acquisition and analysis of important data relating to contractile activity development of cardiac cells, making it a promising tool for optimization in cardiac tissue engineering.

## Introduction

Globally, nearly 30% of deaths are attributable to cardiovascular diseases (Cardiovascular diseases CVDs, 2024), with an estimated 92 million of adult Americans suffering from at least one type of cardiovascular disease in 2017, while 43.7% of the adult population of the US is projected to develop some sort of cardiovascular disease by 2030 (Benjamin et al., 2017). Due to the heart’s limited capacity of self-renewal and its vital role, the ability to regenerate or heal the heart has always been a major research domain (van Berlo and Molkentin, 2014).

Resident cardiac progenitor cells have been demonstrated to exist, although resident heart cardiomyocytes are believed to be the main source of ongoing renewal in normal mammalian myocardial homeostasis as well as after myocardial injury (Beltrami et al., 2003; Senyo et al., 2013; Ali et al., 2014). However, these cells are still a relatively inefficient source for regeneration (van Berlo and Molkentin, 2014). Alternative techniques, involving embryonic stem (ES) cells or induced pluripotent stem (IPS) cells derived cardiomyocytes, either directly injected or as patches affixed to the native myocardium, have been investigated and show promising results (Kawamura et al., 2012; Chong et al., 2014). Focusing on the latter, which we believe allow better control on the newly generated tissue, we propose an integrated imaging tool to help optimize the generation of these patches.

Cardiomyocytes monolayers have been investigated as a therapeutic tool by many groups, leading to the development of techniques to differentiate stem cells towards the cardiomyocyte phenotype, and therefore increasing their potential (Lian et al., 2012). They not only offer the potential for tissue regeneration, but also as an *in vitro* model for drug testing and disease modeling (Zweigerdt et al., 2003; Navarrete et al., 2013). However, current differentiation protocols are still sub-optimal, resulting in cultures with variable yields and properties. Advances in medium composition, mimicking soluble factors secreted by cells, as wells as environmental factors have been shown to play key roles during differentiation and maturation. Higher rigidity culture substrates have been shown to impact cardiomyocyte yield and guide the maturation of already differentiated cardiomyocytes (Arshi et al., 2013). Other groups have also shown that long-term electrical stimulation of newly differentiated cardiomyocyte-like cells increased tissue maturation by improving their structural and functional properties, promoted ventricular-like phenotypes, improved calcium handling and increased cardiomyocytes yield amongst other demonstrable benefits (Chan et al., 2013; Hirt et al., 2014).

Here, we propose a lenseless real-time imaging system to monitor cardiac cell sheet function and structure in culture, providing the necessary tools to quickly evaluate monolayer cultures. This imaging device allows continuous evaluation of contractile properties as well as structural cell organization during culture. Additionally, an electrical stimulation unit is coupled with the imaging system, for long-term and acute stimulation of the tissues. Other groups have previously proposed lenseless imaging set-ups for cardiomyocyte cultures (Kim et al., 2011; Zheng et al., 2011), though none of these systems were as complete for cardiomyocyte cultures as our proposed method where we included electrical stimulation, high-speed imaging and programmable illumination/imaging protocol. We believe that the information the system can provide combined with the appropriate feedback is crucial to develop optimal cardiac tissues for drug screening or engineered replacement tissue.

## Methods

### Prototype design

#### Main components

**FIGURE 1:**
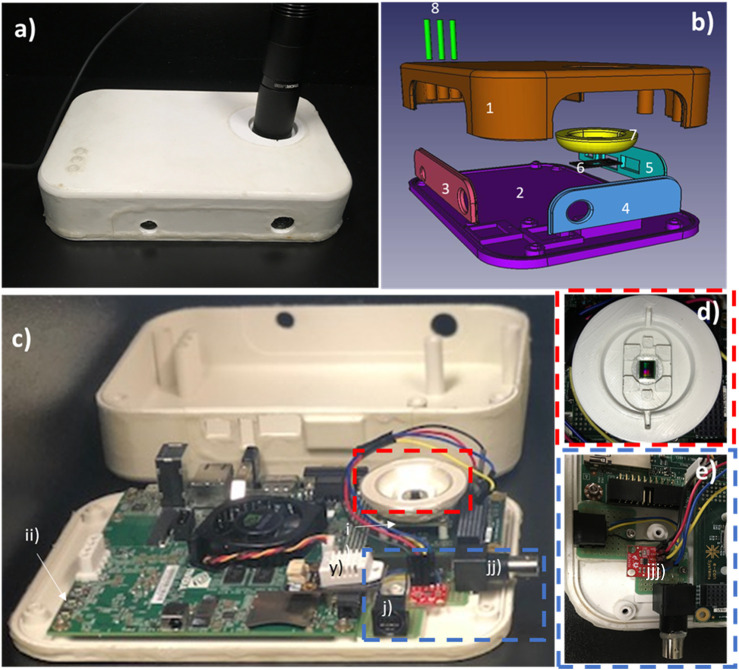
Imaging module design. **(A)** Closed view of the prototype, **(B)** CAD exploded view of the system enclosure designed for the protection of the imaging device, **(C)** open view of the embedded system, **(D)** overview of the culture area and **(E)** zoomed view of the added connectors and circuits. The casing was designed specifically for the purpose of protecting the electronic components from shocks and humidity. In addition to the casing, a thin coat of silicone (Leak Seal, Rust-Oleum) has been applied to both sides of each printed piece. The casing is formed of eight different components that easily “click” together. The top (1) and bottom (2) hold the three side panels (3–5) while parts (6) and (7) are screwed from under the image sensor’s PCB as showed in i). Three copies of part 8 are slid in the top part (1) to allow button controls on the Jetson development board (ii) and are held by the silicone coat. In addition to the already present power, usb3 and ethernet connectors, 9 pins (j) and a BNC (jj) connectors have been added inside the case. The BNC is connected to the DAC (jjj) in order to modulate the input to the LED driver (DC2100, Thorlabs Inc., USA) while the 9 pins connector is only connected to the necessary I2C pins of the Jetson development board (Figure 2E). Additional pins have been left available for future features. A humidity and temperature sensor (y) was fixed with “hot glue” on top of the Jetson.

An imaging device, presented in Figure 1, was developed for real time monitoring and evaluation of tissue cultures. The main processing unit of the system is a Jetson TK1 graphic development board, allowing high throughput frame processing. Using the Jetson TK1 with Ubuntu 14.04, enables the system to behave as a stand-alone device. A screen is connected using a HDMI output, and a wireless mouse and keyboard are connected in the available USB3 port. The Jetson can be connected to the internet.

The selected camera is the e-CAM40_CUTK1 (E-Con System Inc., India) which was the fastest sensor compatible with the Jetson TK1 board available at the time of development. The camera uses a 4-lane MIPI CSI-2 interface to the board. It comes on a mounting board with its drivers, allowing for easy “plug-and-play” with the Jetson board. Since it was supplied with a sample application with limited functionalities, programs were developed to control the camera and acquire sets of images, as detailed in the following sections.

#### Imaging sub-unit

As our system is lenseless, the “shadow-imaging” approach was selected as the imaging technique. Although it does not allow for magnification, its wide field of view and minimal post processing make it a suitable technique for real-time imaging and processing. In brief, a LED (M505L2, Thorlabs inc.), controlled by a LED driver (DC2100, Thorlabs inc, United States), illuminates a pinhole (D610-1500-MD, Thorlabs inc) positioned approximately 5 cm perpendicular to the sample and rests on the imaging sensor. Since the pinhole is distanced from the sample, the light waves can be considered planar and the objects, which in our case are mostly transparent, refract light. As the image sensor is very close (less than 200 µm) the diffraction is minimal, and the images received consist of small rings corresponding to the area of each cell. This lenseless technique is limited by the resolution and size of the sensor. The sensor of the camera is the Omnivision OV4682 RGB IR - 1/3″Optical format CMOS which has a pixel size of 2 µm by 2 µm and an area of 5.44 mm by 3.07 mm. The intensity of the LED can be controlled through the Led Driver either manually or by a low voltage input provided by a digital to analog converter (DAC) (MCP4725, Microchip) wired to the Jetson TK1 and communicated with by I2C (Figure 1a). The gen2-I2C bus is used for communication as it works with 3.3V and is available from the expansion header on pins J3A1.18 and J3A1.20. As the camera relays the pinouts because it occupies the entire expansion header, the wire connections in its own expansion are as follow: SCL - CN1.42, SDA - CN1.47, Vcc - CN1.37, Gnd - CN1.2. For ease of connection, the output of the DAC is connected to a BNC connector (Figure 1jj) (1-1634612-0, TE Connectivity AMP) and the connection between the LED driver and the system is ensured by a coaxial cable.

#### Additional sensors

Two additional features were added to the main unit, a 9-pin connector (MD-90S, CUI INC) and a humidity and heat sensor (SEN0137, DFRobot). The first links the four pins required for I2C communication mentioned above with the electrical stimulation sub-system and will allow further future add-ons. The humidity and heat sensor were mainly used for testing and validation of stability when used in the incubator. It was connected to another workstation by USB for display in the Arduino IDE console. The wiring was passed through the BNC connector hole in the casing.

#### Prototype casing

**FIGURE 2:**
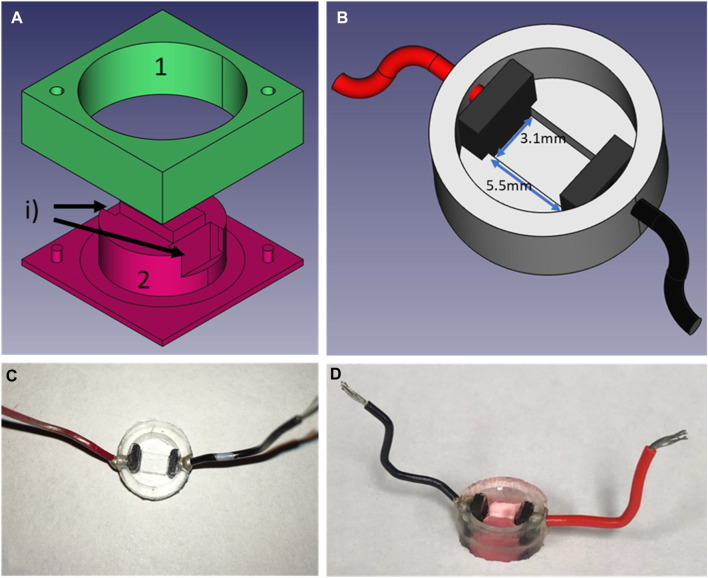
Design of the PDMS culture well. **(A)** CAD exploded view of the mold use to create the wells, **(B)** is the CAD representation of the culture wells with electrodes and wires for field-stimulation, **(C)** is a picture of the wells in PDMS after unmolding and **(D)** the well filled with culture medium and seeded with cells. To create the wells, two carbon electrodes are positioned in slots labeled i) after part 1) of the mold is firmly in place on top of part 2). Liquid PDMS is then poured into the mold. To ensure a fixed height for the bottom of the well, part 1 is 0.1 mm taller than part 2. In addition, a cover slip is placed on top of part 1 to ensure a flat finish. The mold is then placed in the incubator at 37° overnight. After unmolding, holes are punctured on the side of the wells to allow the cold soldering of the wires using silver paste (8331-14G, MG Chemicals) and let to cure for a minimum of 4 h. Before seeding, wells are submerged in 70% ethanol and rinsed with dH2O before passed under UV light for a minimum of 30 min.

**FIGURE 3:**
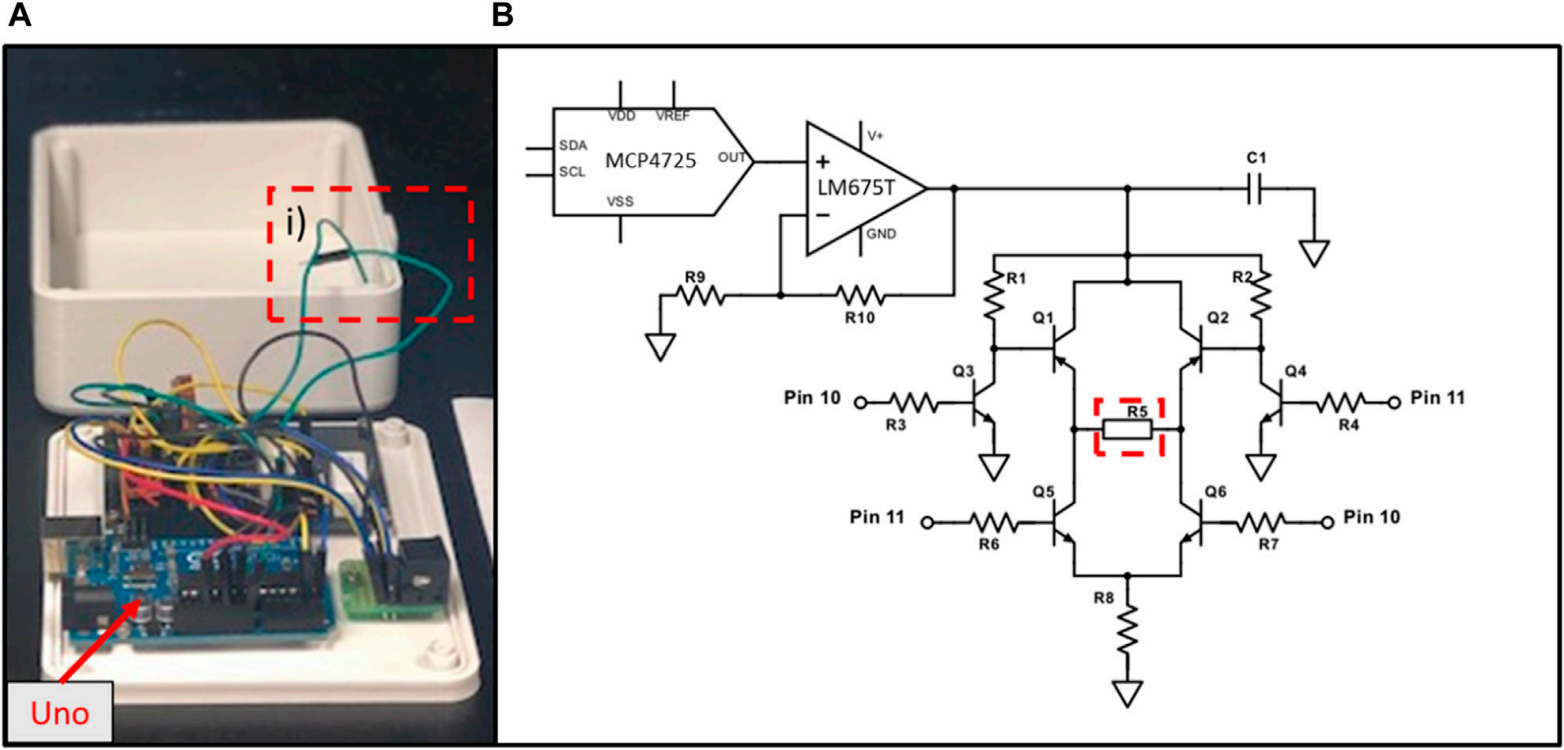
Electrical stimulation module: **(A)** an open view of the prototype; **(B)** schematic of the circuit. The system relies on the same principle as an H-bridge, where “switches” created with transistors (Q1, Q2, Q5 & Q6) are closed in alternation to allow the same current to pass through the tissue (R5). R8 = 50* Ω, is the safety resistance that limits the current to a maximum of 200 mA. The frequency and the duration of the biphasic stimulation is controlled by an Arduino Uno (1), which applies or not 5V to pins 10 or 11. The current intensity is controlled by the imaging unit via a DAC communicating by I2C. The voltage generated by the DAC is multiplied by a factor of 3 using a simple non-inverting op-amp circuit, where R9 = 500 Ω and R10 = 1000 Ω. Capacitor C1 (10 nF) has been placed as close as possible to the input of the “H-Bridge” to minimise the interference noise. Additionally, a heat sink has been apposed to the back of the op-amp to limit heat noise.

**FIGURE 4:**
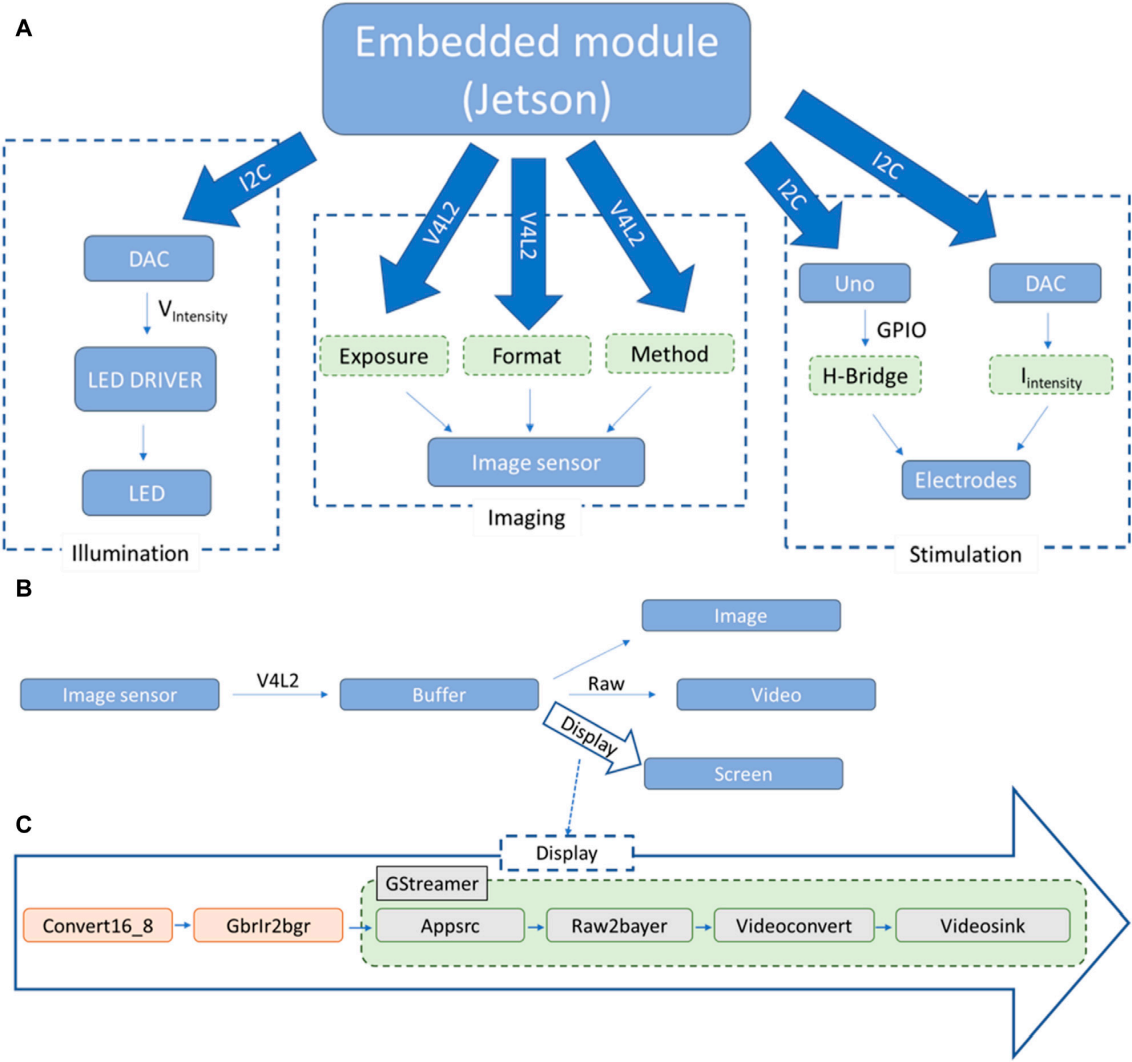
Schematic representation of the high level interactions between the different components of the system: **(A)** downstream relationship between the main module (Jetson TK1, Nvidia) and the principal systems, (sample illumination, imaging and electrical stimulation); **(B)** upwards passage of information from the image sensor to the main module; **(C)** pipeline used for the display of frames on screen. The illumination module relies on the possibility of our LED driver (DC2100, Thorlabs Inc., United States) to be modulated externally by a voltage input. Our program allows communication with a mounted DAC module (MCP4725, Microchip) via I2C communication. With this approach, the LED is turned on only when acquiring images and it is possible to modulate the light intensity when imaging cultures.

**FIGURE 5:**
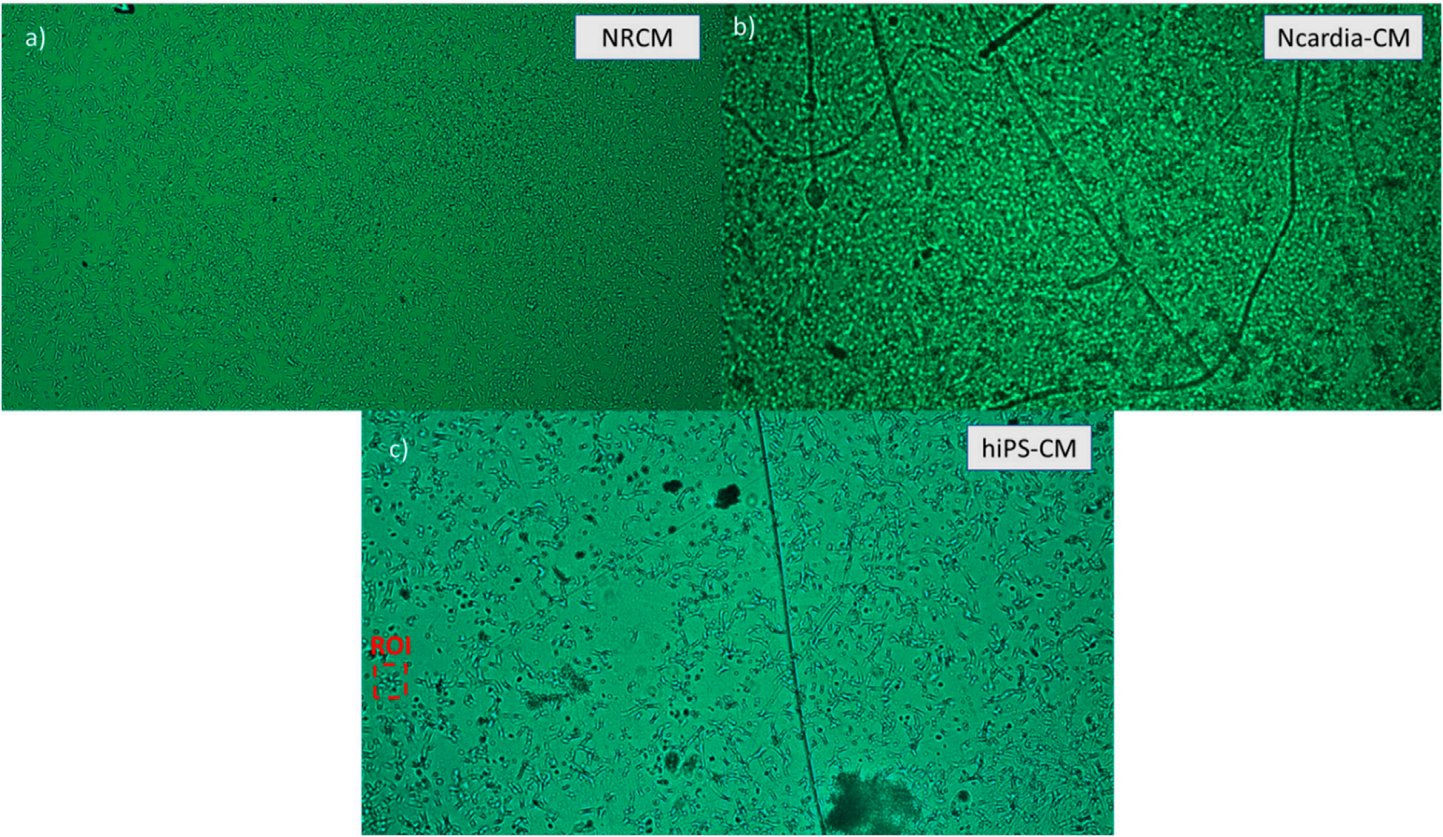
Different cell lines captured and monitored with the device. **(A)** Neonatal rat cardiomyocytes seeded 3 days prior to imaging at a density of 30,000 cells/well (∼1875 cells/mm2). **(B)** Ncardia commercial cardiomyocytes derived from human pluripotent stem cells (hIPSC-CM) seeded 4 days prior to imaging at a density of 50,000 cells/well. **(C)** In house hIPSC-CM, seeded on the PDMS wells 3 days prior to imaging at a density of 5,000 cells/well.

**FIGURE 6:**
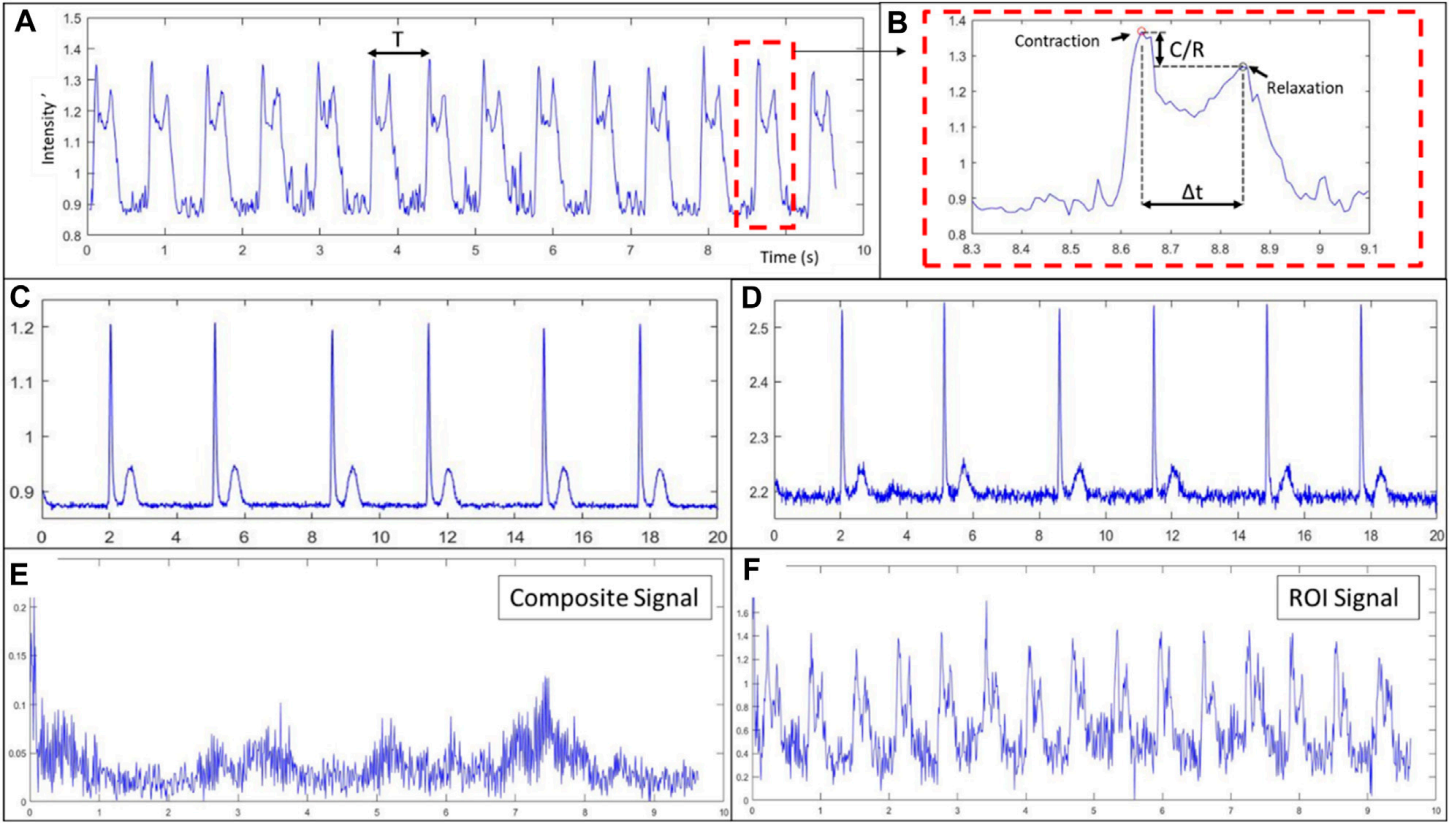
Spontaneous contractile signals computed from videos captured with the imaging device. **(A)** Characteristic composite signal from tissue composed of Ncardia cardiomyocyte derived from hIPSC, **(B)** contraction-relaxation pair zoomed view and contractile characteristics identification. **(C,D)** Characteristic composite signal from NRCM tissue computed with a delay of acquisition of 6 **(C)** or 3 **(D)** between frames. **(E)** Composite signal from tissue of in-house hIPSC derived cardiomyocytes seeded at low density (Figure 5C), **(F)** contractile signal of the region of interested (ROI) (see Figure 5C).

**FIGURE 7:**
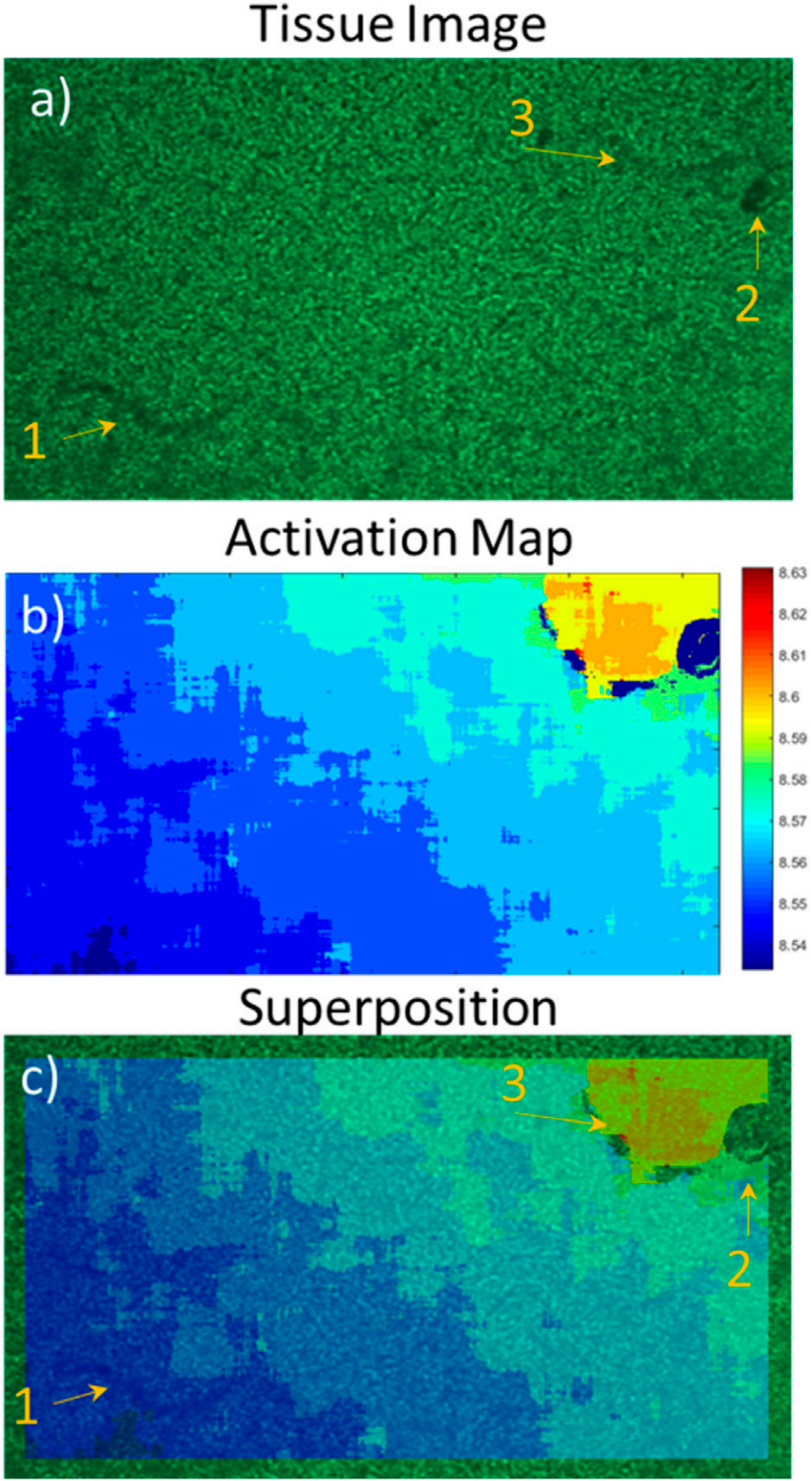
Superposition of the activation map on the tissue image; effect of tissue organisation on contraction. **(A)** Neonatal rat cardiomyocyte image with the imaging device, **(B)** activation map of the first beat from the contractile signal, **(C)** superposition of **(A,B)**. From the activation map, the contraction origin is found to be in the bottom left corner with a linear propagation towards the top right corner.

**FIGURE 8:**
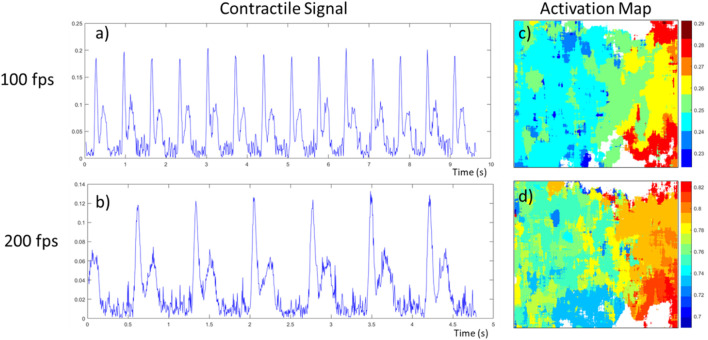
Effect of speed of acquisition (frame per second) on contractile signal and activation map on the same neonatal rat cardiomyocyte tissue. **(A,B)** The contractile signal obtained from the video, at 100 and 200 fps respectively; **(C,D)** the activation maps obtained from the first beat of these videos. The contractile characteristics are the following respectively, beating frequency of 1.47 Hz and 1.39 Hz, time between peaks of 192 ms and 186 ms, relaxation to contraction ratio of 0.521 and 0.563.

To protect the whole system from liquids, small impacts and humidity, we designed a case (Figure 1B) and 3D printed it in Polylactic Acid (PLA) (Polar White, CEL Robox) using a 3D printer (RoboxDual Micro-Manufacturing Platform, CEL Robox) with 0.4 mm resolution. This case is formed of eight different parts interlocked with each other. The top and bottom components have a length of 210 mm, a width of 140 mm, and heights of 39 mm and 6 mm respectively. The bottom part has holes to allow the Jetson TK1 board, the BNC connector, the 9-pin connector and the top part to be tightly screwed in and provides a supporting pad for the imaging sensor. The three side panels are held in place by the top and bottom components and allow access to the systems connectors. These panels have been printed separately from the top piece to facilitate assembly, as some connectors protrude from the casing, and allowing future design modifications. As cell culture is performed on top of the imaging sensor, a circular 100 µm glass slip (12-545-80, Fischer Scientific) is glued to a thin rectangular part (Figures 1B–6) over which a bowl-like component (Figures 1B–7) is positioned to hold it in place. Silicone (Clear Kwik Seal, Dap) is applied at the junction between the two parts to completely seal this protective structure and both are held in place by screws on the image sensor. These parts add no more than the thickness of the cover slip between the sensor and the culture wells. They can be easily replaced if scratched or damaged. Three thin cylinders (Figures 1B–8) are slid in the top part to allow pressing of the Jetson TK1 control buttons and are held in place by silicone. All parts of the casing are sprayed with silicone (Leak Seal, Rust-Oleum) on both sides before assembly, sealing them completely. Once assembled, the casing-to-casing and wire-to-casing junctions are sealed with commercial silicone (Clear Kwik Seal, Dap), protecting the electrical components from the high humidity present in culture incubators.

#### Culture wells

To minimize the light diffraction, it was essential to decrease the distance between the sensor and the sample to a minimum. Since the imaging surface area of our system is no more than 5 mm by 3 mm, we created custom culture wells (Figures 2C, D) with Polydimethylsiloxane (PDMS) (184 Silicone Elastomer Kit, Sylgard), a transparent and widely used silicon-based organic polymer (Lee et al., 2004). These wells have thin bottoms (<100 µm) and a tunable rigidity by selecting the ratio of curing to polymer (Arshi et al., 2013; Boudreau-Béland et al., 2015). These wells are created with our designed two-part molds (a) machined in polycarbonate (Lexan, Sabic Inc.) with a CNC (iModela, Roland) using a Flat End Mill (EMF-125-2F-031, Roland). PDMS is first mixed, then stirred for 5 minutes and degassed before being poured into the mold. Two carbon electrodes (∼4 × 5 × 1.25 mm, SK-05 ISO Graphite Plates, Industrial Graphite Sales LLC) can be placed in the mold (Figure 2A, label i) before curing for tissue electrical stimulation. To assure a flat finish and the exact height of the wells’ bottom, a plastic cover slip is gently placed over the mold, avoiding the formation of bubbles in the process. Once cured, if electrodes have been included, holes are punched on the side of the wells and 1-inch wires are soldered with silver paste (8331-14G, MG Chemicals) to the electrodes. The resulting wells have an outside diameter of 11.9 mm, an inside diameter of 9.0 mm and a height of 4.5 mm, while the primary culture area between the electrodes is of 3.1 mm by 5.5 mm. Before cell culture is performed in these wells (Figure 2B), since PDMS is hydrophobic (Bodas and Khan-Malek, 2007), a 60 s air plasma treatment (PDC-32G, Harrick Plasma Inc) is required to improve hydrophilicity of the membrane. Plasma treatment of the wells helps with adhesion of the cells and was used for all cell types used in this study.

#### Electrical stimulation sub-unit

An electrical stimulation unit (Figure 3A) has been developed for transient as well as chronic stimulation during stem cell-derived cardiomyocyte maturation. This unit is linked to the imaging unit by a cable plugged in the 9-pin connector on both units. As previously mentioned only 4 of these wires are currently used, more specifically 5V, Gnd, SDA and SCL. This unit has a tunable frequency, duration of pulse and current intensity. It is based on an Arduino Uno driving a “H-Bridge”-like electrical circuit. The schematic of the circuit is presented in Figure 3B. Stimulation intensity is modulated using a DAC (MCP4725, MicroChip) connected to a non-inverting op-amp circuit. The resistances create an amplification ratio of 3 (R9 = 0.5 kΩ and R10 = 1 kΩ) while the op-amp (LM675T, Texas Instrument) allows for high current generation. In our set-up, the DAC output is between 0 and 3.3 V, while the supply voltage for the op-amp is 12 V. Although we did not use a rail-to-rail op-amp, the stimulation current is supressed when all switches (Q1-Q2-Q5-Q6) are open. In addition, our circuit allows the passage of current between the electrodes (R5) in both direction with only one current source. The Arduino Uno controls the opening and closing of the switches by applying 5V to its pins 10 or 11. In the electrical circuit, R8 has been divided into two 5 W resistances totaling 50Ω with the purpose of limiting the current through the cell culture to 200 mA. One has been placed where R5 is depicted in the schematic and the other at R8. To get the current to the cell culture well, two wires (Figure 3A, label i) leave from one side of R5 and are connected to the wires soldered to the carbon electrodes. In a similar manner than for the imaging unit, a case has been designed and 3D printed in PLA, consisting of a bottom and top part screwed together. These printed parts are also spray coated on both sides with silicone (Leak Seal, Rust-Oleum) and all junctions are sealed with silicone (Clear Kwik Seal, Dap).

### Programming

All functionalities of the system are controllable by the Jetson TK1 (Figure 4A). Having its own distribution of Ubuntu installed, it is possible to run different programs developed for this project and achieve evaluation and monitoring of cardiac cell cultures. The main programs can be divided into four categories: illumination, imaging, electrical stimulation, and analysis. The code for the embedded system has been written in C, while part of the analysis post-processing is performed in MATLAB (Mathworks, 2018) at this stage.

#### Illumination and imaging

The first two functionalities have been grouped and presented in a graphical user interface (GUI) developed with GTK3+ (GTK+ Team). The Linux library i2c-dev and the system ioctl library are uses to communicate with the DACs. The I2C pins are accessed on an adapter from the user space and the I2C file, once opened, is written to with the “i2c_smbus_write_word_data” function. For the imaging system, the V4L2 API manages the setup and frame grabbing of the imaging sensor and GStreamer the image handling and display. The principal steps for the first part are the following: open the device, set up the exposure, negotiate the frame format and the method used to access the frames, create and allocate the memory for the different buffers, generate a main loop where frames are acquired from the sensor and, when finished with the program, close the device and free the memory. More information on the V4L2 API can be found in the Linux Media Subsystem Documentation (Linuxtv, 2018). Here, the main loop of the code is called by a timer with a duration of 3 ms, the method used is primarily mmap and the frame format is BGGR (BG10) with either 672 × 380 or 2,688 × 1,520 pixels per frame acquired. The theoretical acquisition speed is capped at 330 frames per second (fps) as given by the manufacturer, justifying our use of a timer every 3 ms. It is worth noting that in practice the frame rate is going to be limited by the selected exposure. In the main loop, each grabbed frame is put in a buffer from which three actions can be done simultaneously (Figure 4B). If a video is being recorded, each of these frames is temporarily placed in a larger buffer until enough frames have been acquired and the whole buffer is written to a binary file. This method allows for high-speed acquisition and conservation of the entire information contained in the frames. To display the grabbed frames on screen, one frame every 60 ms is pushed to the GStreamer pipeline presented in Figure 4C. The frame format, specific to the CMOS sensor, is 10 bits raw BGR-IR. Instead of a second green pixel as in regular BGR format, it acquires infra-red (IR) intensity, and each color pixel is stored in a 16 bits word where the 6 most significant bits are unused. For simplicity and to facilitate the use of pre-existing GStreamer elements, pre-processing of the frames is performed before feeding them to the Appsrc element: Replacing the IR pixel with green values and converting the 16 bits per pixel to 8 bits. To save images to memory, a similar pipeline to the display one has been created with a mpeg and filesink element replacing the videosink element present in Figure 4C.

#### Electrical stimulation

A custom communication protocol using the same I2C technique presented above has been developed for the communication between the Jetson and the Arduino Uno, which acts as a slave to the imaging unit presented in Figure 1. A command such as the “frequency” is sent first, then the desired value is sent in the next message. On the Uno side, it is constantly listening for new commands and once it receives one, does the appropriate changes in its stimulation settings. Electrical stimulation requires the start command to begin, as it is by default in the stopped state. Communication with the DAC is done as for the illumination system, calling 0 × 61 instead of 0 × 60 for the DAC’s address. In both cases only the 0 × 40 registry is written to, updating only the DAC register and not the EEPROM.

#### Real time analysis

The analysis is performed in two steps. The first step is done while the signal is acquired, whereas the second is done in post processing on a workstation. The two follow the same logic to get the contraction rate of the tissue and is based on our published algorithm (Béland et al., 2022). The idea is to get the variation rate of light intensity per pixel, as captured by the sensor. Since the LED intensity is kept constant, the only thing affecting the intensity received by the sensor is the cells contraction. To calculate the rate, we subtract the total frame intensity of two frames and divide by the time separating the two. As the number of frames per second acquired is known, the only parameter that needs to be set is the delay between frames (τ), which we set at 6 times the fps divided by 100, based on empirical analysis. For a video with an acquisition speed of 200 fps, this means the delay between frames would be of τ = 12 frames. The equation below is therefore used to acquire the composite signal:

$$\Delta S\left(t\right)=\frac{1}{N_{x}N_{y}}\sum_{j=1}^{N_{y}}\sum_{i=1}^{N_{x}}\left|\frac{M_{i,j}\left(t\right)-M_{i,j}\left(t-\tau \right)}{\tau }\right|$$ (1)

This derivative like algorithm gives a continuous signal characterised by two peaks, one of which is associated to the contraction of the tissue and the other to the relaxation (Figures 6A, B). The contraction peaks are found automatically using an algorithm which uses an adaptive threshold, first derivative sign change and a minimum peak width. From the time between two contraction peaks, we deduce the beating rate of the tissue and display it in real time. The obtained signal and the detected peaks are plotted in an allocated zone of the GUI using the Cairo library.

#### Post processing

The post processing consists of creating movie format files from the binary files acquired with the camera, getting more information from the composite signal described above and creating activation maps of contractions. The first is either done on the embedded system, by passing the frames read from the binary files to a GStreamer pipeline, or with MATLAB on a separate workstation. This GStreamer pipeline has the same elements as in Figure 4C, but with a video encoder and file sink elements instead of the video sink. In MATLAB, this task is performed using the *videowriter* object and the *raw2rgb* functions. In addition to the beating frequency, we propose to look at the ratio between the contraction and the relaxation peak and the time between the two. We believe these signal characteristics are an important source of information as they are directly impacted by physiological phenomenon. For example, if the action potential duration is increased by lower temperatures, the time between the contraction and relaxation peak is expected to increase. To get these signal characteristics, the peaks are found in MATLAB using the *findpeaks* function with custom input arguments and the characteristics computed automatically. The final postprocessing task is the creation of activation maps. These maps allow to determine the propagation speed of the contractile activity and to evaluate the ectopic stability. To achieve this, a 31 by 31 window is first passed over each frame and the contractile signal, the speed of light intensity variation, is computed for each pixel. All contraction peaks are found, and each beat is isolated. Maps are then plotted where the time at which each pixel has contracted is color-displayed.

### Validation

To test the different functionalities of the device, different cardiomyocytes cell lines were used: neonatal rat cardiomyocytes (NRCM), commercial (NCardia-CM) and non-commercial human induced pluripotent stem cell derived cardiomyocytes (hiPS-CM). Each one was cultured according to their respective protocols, as detailed below. The validated functionalities with all three cell types were the capacity to acquire clear images of the cultures, obtain videos of contracting tissue, compute the contractile signal from these videos and creating the activation maps. Electrical stimulation validation was performed only with the NRCMs. All acquisitions were done in a O_2_ Control *In Vitro* Glove Box (Coy Laboratory Products, Inc., United States) with 20% O_2_ level and 5% CO_2_. N_2_ was used as the 3rd gaz to obtain the O_2_ and CO_2_ levels.

#### Neonatal rat cardiomyocyte culture

Animal handling procedures were conducted according to the Canadian Council on Animal Care guidelines and were approved by the Montreal Heart Institute Animal Research Ethics Committee. Beating hearts were removed from sacrificed Sprague-Dawley rats, aged one to 3 days old, and kept on ice in Ca^2+^ and Mg^2+^ free Hank’s balanced salt solution. Ventricular muscle tissue was excised and cut into 1–3 mm^2^ pieces. Purification was performed over night using a Neonatal Cardiomyocytes Isolation System (Worthington Biochemical). In parallel, PDMS wells were immersed into 70% ethanol and rinsed with distilled water, plasma-cleaned for 1 min (PDC-32G, Harrick Plasma Inc.) and then sterilized under UV light for 1 h. Following purification, cells were seeded in the prepared PDMS wells at a density of 30,000 cells per well and maintained in Dulbecco’s Modified Eagle’s Medium (DMEM, SLM-220-B, Millipore) with 10% fetal bovine serum (FBS, ES-009-B, Millipore) and 1% penicillin/streptomycin (TMS-AB2-C, Millipore). Culture wells containing the cells were placed either on the sensor or in a 10 cm petri dish and in the incubator at 37°C and 5% CO_2_. Imaging was performed 3 days after seeding.

#### Commercial hiPS-CM culture

Cor.4U^®^ Human iPS Cell-Derived Cardiomyocytes (NCardia, Germany) were thawed as described in the manufacturer’s protocol and maintained in Cor.4U^®^ complete medium as previously described (Gélinas et al., 2017). hiPSC-CMs were thawed and plated onto 0.1% gelatin coated 12-well plate (2 × 10^5^ cells per well) and culture medium was replaced every 3 days until analysis (less than 60 days). hiPSC-CM were then dissociated by incubation with TrypLE Express (Gibco) at 37°C for 10 min before being plated directly onto the gelatin coated PDMS wells (10 × 10^4^ cells per well). Spontaneously beating cells were seen 2 days after being plated. Imaging was performed 2 days after observing spontaneous activity.

#### hiPS-CM culture

Commercially available hIPSC (CW200-47, CIRM) were differentiated to hIPSC-CM using a small molecule modulation differentiation protocol followed by a glucose starvation as previous published by Sharma A. *et al.* (Sharma et al., 2015). Approximately 100,000 hiPSC were plated on hESC qualified Matrigel (Corning) coated 6-wells culture plate. Once 85% confluency was reached, hiPSC were incubated with 6 μM CHIR99021 (Sigma) in RPMI/B27 minus insulin (Life Technologies) for 48 h. After 48 h medium was changed to RPMI/B27 minus insulin until day 3 when medium was replaced by RPMI/B27 minus insulin with 5 μM Wnt inhibitor IWR1 (Sigma) for 48 h. At day 5 medium was changed to RPMI/B27 minus insulin for 2 additional days. At day 7, medium was replaced with RPMI/B27 (with insulin). Spontaneously beating hiPSC-CMs were observed between day 7 and day 10. At day 10, medium was changed to low glucose RPMI/B27 and cells were maintained in this medium for 3 days. At day 13, cells were washed with DPBS to inhibit contraction and cells were dissociated using TrypLE Express (Gibco) with 0.5 U/mL liberase TH (Roche) at 37°C for 10 min. Cells were then diluted in 5 mL RPMI/B27 before being centrifuged. Cells were resuspended in 1 mL RPMI/B27 and passed through a 100 μm cell strainer before being plated directly onto the Matrigel coated PDMS wells (5 × 10^4^ cells per well). Contraction was seen after 3–5 days.

Commercial and hiPSC-CM derived in-house were characterized as described in reference (Gélinas et al., 2017; Gélinas et al., 2024), respectively. Briefly, for each cell type, we showed absence of signal for stem cells markers (e.g., NANOG, POUF5F1), as well as increased expression of cardiac marker genes, such as cardiac transcription factors (NKX2.5 and GATA4) and/or structural elements (TNNT2 and MYH7). In addition, in terms of immunohistochemistry, cardiac phenotype has been confirmed by staining of α-actinin and/or cTNT.

## Results

Each module of the system was first validated individually, while the complete system was validated using all three cardiomyocyte cell types.

### Prototype assembly

The imaging sub-system is presented in Figures 1A, C, while the electrical stimulation sub-system is presented in Figure 3A. Once assembled, Ubuntu 14.04 as well as all the required programming libraries and drivers were installed on the Jetson TK1. Validation of the imaging system was achieved by acquisitions of images of random shapes placed on top of the sensor. All connections were tested using a multimeter and sealing was tested using the humidity and heat sensor, with the prototype inside a conventional incubator at 100% humidity. Although some humidity could infiltrate the casing, humidity level inside the case did not rise above 70% in a 10-h monitoring period. Evaluation of the illumination system was performed using the imaging sensor as a measuring tool for light intensity. The system was deemed validated once light intensity was fully tunable by the main unit. The current intensity driving the LED was fixed to a range of 0–200 mA, corresponding to an input voltage range of 0–3.3 V.

### Cell culture

All cell cultures were performed according to the protocols presented above and in accordance with good manufacturing practices (GMP). For the cardiomyocytes derived from stem cells, the phenotype was deemed achieved when tissue contraction was observed. In neonatal rat and NCardia’s cardiomyocytes, close to 100% of the tissue were considered to be cardiomyocytes, while the hIPSC-CM were close to 100% differentiation following purification. An image of cardiomyocytes cultured in a PDMS well is presented in Figure 2D.

Each well was unmolded and tested for leaks with dH_2_O, then submerged in 70% ethanol before being sterilised under UV light for a minimum of 1 hour. For neonatal cardiomyocytes, wells were plasma-cleaned before being sterilised by UV.

### Tissue imaging

#### Acquisition of images of the different cell types

A sample image of each cell type is presented in Figure 5. Exposure was set to 10 ms, the input voltage for light intensity was set to 1.65 V and the frame size was 2,668 × 1,520 pixels. In Figure 5A, neonatal rat cardiomyocytes (NRCM) are imaged 3 days after seeding in the PDMS well. While it is possible to easily see cell contours, 100% confluency is not achieved in this image as cells would have required additional days to form a fully connected tissue. In Figure 5B, NCardia’s commercially available hIPSC derived cardiomyocytes are imaged 4 days after seeding. The long darker lines present in the image are due to scratches and debris on a mold piece. Tissue growth is good in this case as most cells are touching their neighbors. Individual cells are hard to distinguish within the tissue as compared to a lower density. In Figure 5C, a monolayer of cardiomyocytes derived in house from hIPSCs is presented. Seeding was of 5,000 cells per well, allowing for clearly distinguishable cells. By doing this we validated the possibility for individual cell monitoring. In summary, these high-resolution images provide information on the tissue organisation opening to the possibility for spatial pattern analysis.

#### Contractile signal from videos

Videos of 10–20 s, with acquisition speed ranging from 100 to 300 fps, were recorded. Led intensity was adjusted as the exposure of the sensor was changed to reach high fps. Contractions could be observed in real-time, confirming that the tissues were usable for our analysis purposes and validation. Contractile signals for each cell type are presented in Figure 6. In Figure 6A, we show the signal obtained from a tissue of commercial cardiomyocytes derived from hIPSCs. The contraction and relaxation peaks observed are due to the spontaneous activity of the tissue. As an example of the signal characteristics derived from such signal, the average characteristic parameters of this tissue were the following: a period (T) of 710 ms, a time between contraction and relaxation (Δt) of 200 ms and a contraction intensity over relaxation intensity ratio (C/R) of 0.94. In Figure 6C and d the signal is obtained from tissue of neonatal rat cardiomyocytes. Figure 6E shows the signal from a tissue of cardiomyocytes derived from hIPSCs while Figure 6F shows the signal from in-house hIPSCs derived cardiomyocytes.

The stability of these parameters was evaluated within the same acquisition and over time while keeping the environment stable (data not shown). Four video recordings of 10 s of the same NCardia-CM tissue, taken 30 min apart, were used for this analysis. All three characteristics were stable. Within the same recordings, the highest standard deviation measured was of 0.090 for a period of 1.35 s, 0.026 s for a delay between peaks of 0.340 s and 0.034 s for a peak ratio of 1.243. Over time, the means of each of the average characteristics from each video had standard deviations no higher than 0.01, meaning the measurements were reproducible if the environment was kept stable.

Signals obtained from different wells containing same species tissue were compared in regards of the contractile characteristics dependent on the spontaneous beating period. For this, we used seven different wells containing neonatal rat cardiomyocytes. The seeding density was of 1.9 × 10^5^ cells per cm^2^. We recorded videos 3 days after seeding, each video lasting 10 s with acquisition speeds of 100 fps. Four of these tissues had beating rates higher than 2 Hz, while 6 had rates higher than 1.5 Hz. Only one had a lower beating rate, which was of around 0.33 Hz. No exact relations could be deduced from these measurements, although a trend was observable; time between contraction and relaxation decrease as the beating rate increases while the peak ratios increase with the frequency.

In Figures 6C, D, we show the impact of the delay between frames, “τ” in Eq. 1, on the signals obtained from the same video recording of spontaneous activity of neonatal rat cardiomyocytes. In Figure 6C, we used a delay of 6 images (with 100 fps), meaning the time between frames is roughly 60 ms, whereas in Figure 6D, we used a delay of 3 images, resulting in a 30 ms delay. As it is observable when comparing the two, Figure 6D has a noisier signal which can lead to harder peak detection, as it can be observed in the first relaxation peak. On the other hand, having a smaller delay means the speed of variation of light intensity is closer to the instantaneous change and the obtained signal is more representative of the contraction speed of the tissue.

#### Activation maps

Activation maps of the different tissue types were produced from recordings. An activation map made from the signal acquired from a neonatal rat cardiomyocyte sheet is presented in Figure 7B, with an image of the tissue (Figure 7A) as well as their superposition (Figure 7C). Activation maps allow finding the origin of contraction as well as the stability of the ectopic center. By looking at Figure 7B, we can deduce that the origin in this case is in the bottom left corner of the tissue. Superimposing the activation map with the image gives more insight into the structural dynamics of the tissue as we can determine where connections are present and which regions contract. Using Figure 7, we observe that markers 1 and 2 appear as darker objects in the initial image (Figure 7A) than marker 3. The effect of those tissue “defects” on its contractile capabilities are not what we had expected since only the zone of marker 3 seems to have a direct impact on the propagation, acting as a blocker of contraction. It is possible zone 2 impacts the cells above it, though it is lower than the circular region without contraction. Zone 1 does not seem to impact the tissue contraction, meaning it was probably just a defect on the outside of the well. Also, the activation maps allow to approximate contraction propagation speeds. In the case of the tissue presented in this figure, we can observe a propagation from bottom left to the top right, meaning an approximate distance of 6 mm, in around 60 ms. This corresponds to a propagation speed of 100 mm/s. Of note, the difference in size between the activation map and the image is due to the algorithm creating the map because only pixels covered in full by the convolution matrix (31 × 31 pixels) are kept (no zero-padding was used for border pixels).

In addition to validating the prototype’s capacity to generate these activation maps, we explored the effect of the acquisition speed on both the contractile signals and activation maps. For this, we used the same tissue preparation of NRCM, and took two acquisitions of 975 frames, one at 100 fps the other at 200 fps. The resulting signal and activation maps for their first contraction are presented in Figure 8. The contractile characteristics computed from the videos are as follow, respectively: a beating frequency of 1.47 Hz and 1.39 Hz, a time between peaks of 192 ms and 186 ms, and a relaxation to contraction ratio of 0.521 and 0.563. As these videos were taken in an environment where small variations in temperature are possible over time, this could explain why the frequency reduced for the second recording at 200 fps. As the beating rate is the same, using a higher sampling speed allows the contractile signal, more specifically the contraction peaks pair, to have twice as many data points, meaning a better time resolution. As an example, in Figure 8B, we can observe a slight tilt of the relaxation peak towards the right whereas in Figure 8A it is uncertain if it is not just due to noise. Additionally, this better time resolution permits the activation maps to have more time increments, as shown in Figures 8C, D.

### Electrical stimulation

**FIGURE 9:**
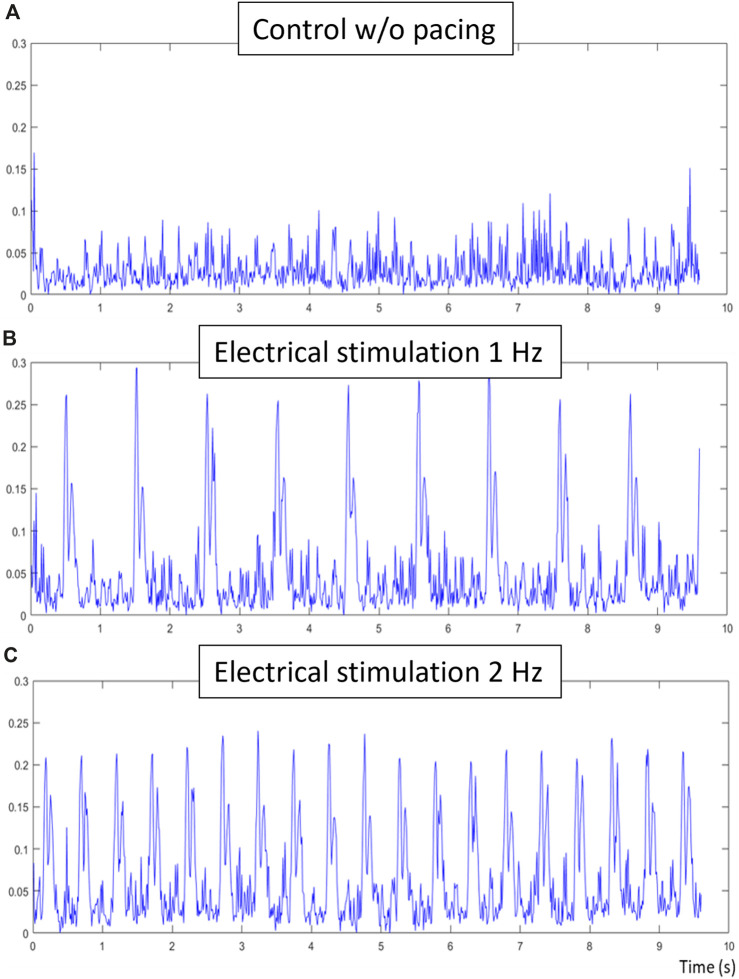
Electrical stimulation validation, **(A)** is the control composite signal obtained without electrical stimulation of the tissue, **(B)** is the composite signal obtained during 1hz electrical stimulation of the tissue and **(C)** is the composite signal obtained during 2 hz electrical stimulation. The same sample has been used for all three acquisitions, each of 10 s at 100 fps. While no contractions were detected in the control signal, clear contraction-relaxation pairs can be observed in the electrically-stimulated signals. Respective contractile characteristics of these signals are the following: period of contraction of 1.01 and 0.508, time between peak-pairs of 0.079 s and 0.077, and relative ratio of peak-pairs of 0.91 and 0.95.

The electrical stimulation sub-unit has been tested separately with an oscilloscope and a 5 W resistance of 15 Ω, confirming the biphasic generation of stimulation pulses with tunable frequency, duration of pulse and amplitude through the main imaging unit. Once tested, cell cultures in wells containing electrodes were connected to the stimulation unit. In Figure, three contractile signals are presented of NRCM tissue, initially without uniform tissue contraction. In Figure 9A, the tissue is connected to the stimulation sub-unit, but no pulses are generated resulting in a noisy signal without definite contraction peaks. The tissue has many areas contracting separately, which results in smaller and varying peaks. In Figures 9B, C, electrical pulses of 1 Hz and 2 Hz frequency respectively are applied. The contraction period is equal to the stimulating period with a small difference of a few milliseconds.

## Discussion

We proposed a novel imaging system for continuous evaluation of cardiac tissue, integrated with electrical stimulation and modulable culture substrates. This stand-alone device is capable of imaging and recording videos of cardiomyocyte cultures, reaching acquisition speeds up to 300 fps and computing characteristic signal of the tissue contraction. The stimulation unit can generate a uniform contraction of the tissue enabling stimulation protocol that could allow further analysis of the tissue as well as long term stimulation protocols for cardiomyocyte maturation after differentiation. Activation maps, providing information on the ectopic origin and contraction propagation speed, are computed from the contractile signal. This imaging tool combines many important systems to allow a novel approach to evaluate and monitor cardiomyocyte cultures.

The purpose of this tool, in addition to being a cardiac tissue monitoring and evaluation tool, could be stem cell differentiation protocol optimisation. As an evaluation tool, the characteristic signal gives rapid insight of the contractile properties of the cells. This could be used to evaluate genetic mutations in cardiomyocytes derived from patients or induced mutations by transfection. Currently, methods such as patch clamp or microelectrode array (MEA) do not allow such quick and simple evaluation. Additionally, quick evaluation of contractile function, in combination with the possibility to run multiple experiments in parallel make this imaging system a promising tool for drug screening. In the optic of optimising cell cultures, real-time imaging systems allow the possibility to extract information, in this case contractile function, at any given time of the culture process. While the cardiac differentiation process is quite slow in comparison to an action potential, the imaging device can be used to follow slower cellular phenomenon, without impacting the cultures environment. To this intent, the device can be programmed to acquire 4 megapixels images at long intervals to follow the development of the tissue and its structural organisation. Once contraction would begin, faster acquisition could be acquired alternately. The contractile properties and the structural information could in turn be used to optimise current protocols or develop new techniques that could complement the current protocols used, such as electrical stimulation during cardiac differentiation.

### Acquisition speed

As shown in the result section, higher acquisition speed is crucial to obtain reliable tissue contractile information. In the proposed system, the speed of acquisition reaches 300 fps, with a spatial resolution of 672 × 380 pixels and a pixel size of 8 µm. This spatial resolution allows the creation of more precise data of the rate of variation of the light intensity which is directly correlated to the contraction speed of the tissue. An average action potential, the phenomenon that induces calcium release leading to cellular contraction, would last about 200 ms depending on the beating frequency and is on average less than 250 ms in the case of human cardiomyocytes derived from stem cells (He et al., 2003). To be able to detect small variation in the contractile signal and correlate these changes with the action potential, the more data points available the better. For this reason, our prototype was designed to achieve high acquisition speeds, thus allowing up to 60 data points during a 200 ms action potential.

Additionally, for a culture area with a maximum length of 5.1 mm and a given speed of contraction propagation of 100 mm/s, the maximum time needed for the wave to travel from one end of the tissue to the other is 51 ms. The resolution of the activation maps produced, is limited by the acquisition speed of the system, in this case 300 fps. This results in a maximum resolution of 15 frames. In the case where the propagation is along the width of the culture area (3.1 mm), the maximum resolution drops down to 9 increments. However, if the acquisition speed is lower, for example 60 fps, the resolution would be only 3 frames along the longest direction, further demonstrating the need for a high-speed imaging device. The focal point could be inferred with higher precision using a method similar to the one published by Ponard et al. (2007).

### Electrical stimulation potential

The rational for having a modulable and fully automated electrical stimulation subsystem comes from the need to create standardised evaluation protocols as well as from the reported benefits of long-term electrical stimulation of cardiomyocytes derived from stem cells.

In the first case, it is reasonable to think that the proposed contractile characteristics are highly impacted by the beating rate of the tissue as calcium transients and contractile dynamic are modified by the rate of electrical activation. For this reason, to have comparable results from one experiment to the next, the beating frequency needed to be controllable. However, the electrical stimulation at 2 Hz of a tissue already beating at a 1.9 Hz frequency compared to one beating at a 0.5 Hz is not expected to have the same impact on the contractile properties and in turn the acquired characteristic signal. Additional data needs to be acquired to propose a proper evaluation protocol using the imaging system.

In the literature, experimental electrical stimulation protocols have already been reported. Long term electrical stimulation, for a period longer than 72 h for example, does not seem to affect cell viability, but enhances cell elongation, preserves contractile function, accelerates cardiomyocyte growth and increases their RNA accumulation (Berger et al., 1994; Johnson et al., 1994; Au et al., 2007).

Cardiomyocyte-like cells derived from stem cells have been shown to achieve a more mature phenotype with the use of a long-term electrical stimulation (Chan et al., 2013; Hirt et al., 2014; Ma et al., 2018). Hernández et al. (2016) have published that even single brief periods of electrical stimulation can promote cardiogenic potential of hiPSCs in terms of the number of beating EBs and gene expression of cardiac transcription factors and contractile muscle proteins. By combining a continuous electrical stimulation with the proposed device, the goal is to allow monitoring of maturing tissues and its process, allowing a better understanding. As both acute and long-term seem to have a positive impact on cardiac cells, future work using the system could investigate the optimal electrical stimulation periods.

### Stand-alone vs. scalability

At the core of the system is the Jetson TK1, a stand-alone development card that has the NVIDIA Kepler GPU. The current software does not take advantage of this feature, i.e., parallel programming, as most of the most demanding code, such as the calculation of the activation map, is being performed in post-processing. Having the potential to parallelize code and do heavy processing in real-time is a great feature that could be included in future development of the device. The Jetson TK1 can run as a stand-alone unit which is part of the reason why it was chosen for the system. It is also possible to have it run in remote access from a host computer running Ubuntu. This second option is interesting since it would allow multiple units to be controlled by the same machine and run experiments in parallel as multiple units could fit in a standard culture incubator. The scalability of the system is not dependent on having one host controlling multiple units, though the simplicity of this option is superior. Running multiple experiments in parallel is interesting especially while working on drug screening.

### PDMS wells potential

Another way to run multiple experiments in parallel is the one presented in this work, which is to culture multiple wells at the same time and image each one within a short period of time. This technique limits the monitoring as there is no continuity of the image acquisition but allows many samples to be evaluated with the same imaging system. Each well is made from PDMS, a familiar low-cast biocompatible polymer. In addition to the capacity to control its rigidity, which in turn affects the efficiency and becoming of the stem cell cultures (Arshi et al., 2013), teams have shown the possibility to modify its surface to create micro grooves or patterns (Kim et al., 2012; Dahlmann et al., 2013). These structures impact cell organisation, promoting cell alignment if the micropattern groove are parallel for example, and allow for better reproduction of myocardial architecture and synchronized contraction (McDevitt et al., 2002). Using well patterning would make the cultures even more suited for drug screening and toxicity. Furthermore, having a modulable substrate, the imaging system reinforces its purpose as a tool for cardiac patch optimisation. In addition, using the same molds as presented in Figure 3, one could also create culture wells out of different polymers with superior rigidity.

### Device purpose in the field

Cell based biosensors, more specifically digital movie analysis techniques, are promising for the cardiac tissue engineering field as they offer new ways to evaluate tissue function. The most used of these techniques is videomicroscopy, which has been used to evaluate cardiac tissue since at least 1988, with the work by Eppenberger (Eppenberger et al., 1988). To our knowledge, while beating rate has been evaluated through digital movie analysis since 1990 (Rohr, 1990), no other group has proposed easily evaluable contractile characteristics, while also integrating components specific to cardiomyocyte monitoring, such as electrical stimulation and real-time imaging. Videomicroscopy requires the use of fast acquisition cameras, mounted on optical setups which are not only costly but also, for most, not suitable for use in an incubator. It is advisable to use specialised culture chambers to keep the engineered tissue at the correct temperature and controlled CO_2_ concentration to have reproducible results if the experiments are performed outside the incubator. Our system also includes a software that allows direct monitoring of contraction. To achieve the same results as with our proposed imaging system, one would have to buy and combine many different components, modifying an incubator to accept the camera, cool it, protect it from the high humidity, and develop its own software. An advantage of videomicroscopy over our current setup is its versatility on the acquisition speed and on the spatial resolution.

Other groups have previously proposed imaging techniques for lenseless cell monitoring (Kim et al., 2011; Zheng et al., 2011), which also provide a large field of view and simple optical setup. The system closest to ours was published by Kim *et al.* and used a CMOS sensor from a commercially available webcam and a similar detection system, using light variation peaks as a marker of contraction. Their work allowed for beat detection and beat-to-beat variability measurements, though their system could only reach acquisition speeds of 29 fps and did not offer the possibility of high-resolution images. We believe that for cardiac monitoring, high acquisition speeds are a must since cellular contraction is a rapid phenomenon, especially if one is interested in other characteristics then bpm. However, their work has the advantage of being easier to reproduce as fewer components are required. Another group has proposed an imaging system also using a lenseless technique, subpixel perspective sweeping microscopy, achieving higher image resolution (Zheng et al., 2011). Using their system coined the ePetri, they imaged immortalised cell lines, embryonic stem cells, viral plaque and waterborne parasites, achieving a microscope quality (Zheng et al., 2011; Han and Yang, 2014; Lee et al., 2014). Although not stand-alone, their system allows to look at slow cellular phenomena and is meant to be used in the incubator. The main difference with our system is in the acquisition speed and the purpose of the device, ours being focused on cardiomyocytes while theirs is for cells in general, which explains why our proposed system integrates other sub-systems and high acquisition speeds.

### Comparison of our proposed imaging approach and MEA systems

In comparing our imaging approach to MEA based system, several key advantages emerge. A major limitation of MEA systems is their consumable nature; the microelectrodes deteriorate over time, leading to changes in impedance and signal degradation. This necessitates frequent replacement, with each plate costing ∼US$470 [Axio Biosystems Cytoview MEA 24-well plates, pack of 5 at US$2,337^1^ (Cardiovascular diseases CVDs, 2024)] significantly increasing the overall experimental costs as the number of samples grows. Additionally, each sample must be grown on the MEA and carefully cleaned after each experiment, with uncertainty surrounding the number of times a plate can be reused. In contrast, our approach involves imaging samples directly with cells cultured on low-cost PDMS wells, which can also be adapted for use with glass bottom culture plates at a cost of less than $30 per plate, representing a substantial cost difference.

Another significant difference is in spatial resolution. Our method leverages the high resolution offered by modern CMOS imaging modules, which now boast millions of pixels. Specifically, we utilize the Omnivision OV4682 RGB IR sensor with a pixel size of 2 µm by 2 µm and an area of 5.44 mm by 3.07 mm, providing a distinct advantage in spatial resolution. MEA systems, such as those offered by Axion Biosystems, feature up to 768 electrodes in a single well, though this number decreases in multiwell plates. However, MEA systems do offer superior temporal resolution, with acquisition rates easily reaching the kHz range, which is a trade-off for the higher spatial resolution provided by our imaging approach. Moreover, MEA systems can effectively record the bulk of the tissue’s activity near the electrodes, even in multilayered tissues.

In its current configuration, our system is designed with illumination from one side of the substrate and the CMOS sensor positioned on the opposite side. For multilayered engineered heart tissues (EHTs), this setup may lead to decreased luminosity and spatial resolution due to increased light dispersion. To mitigate these effects, future evaluations could incorporate a near-infrared (NIR) light source and a corresponding NIR CMOS sensor, potentially enhancing the system’s applicability for thicker tissue monitoring.

## Conclusion

In summary, our system is specifically designed for cardiomyocyte monitoring and evaluation, allowing optimisation of future tissue engineering studies. Three different cell types were used to validate the system and all its functionalities, such as electrical stimulation, high-resolution image acquisition, high-speed video acquisition and computing of tissue contractile characteristics. Further investigation is required to determine either species specific markers in the contractile signal or standard characteristics, for the purpose of protocol optimisation, evaluation of mutations induced cardiomyocytes derived from IPSc, drug screening and cardiotoxicity.

## Data Availability

The raw data supporting the conclusions of this article will be made available by the authors, without undue reservation.

## Footnotes

1
https://www.fishersci.com/shop/products/NC1625896/NC1625896, accessed on June 2024.
